# Calcium-based phosphate binder use is associated with lower risk of osteoporosis in hemodialysis patients

**DOI:** 10.1038/s41598-021-81287-4

**Published:** 2021-01-18

**Authors:** Hiroko Hashimoto, Satomi Shikuma, Shintaro Mandai, Susumu Adachi, Shinichi Uchida

**Affiliations:** 1 Department of Nephrology, Shuuwa General Hospital, 1200 Yaharashinden, Kasukabe, Saitama 344-0035 Japan; 2 Department of Nephrology, Graduate School of Medical and Dental Sciences, Tokyo Medical and Dental University, 1-5-45 Yushima, Bunkyo, Tokyo 113-8519 Japan; 3 Department of Cardiology, Shuuwa General Hospital, 1200 Yaharashinden, Kasukabe, Saitama 344-0035 Japan

**Keywords:** Renal replacement therapy, Epidemiology, Osteoporosis, Risk factors

## Abstract

Loss of bone mineral density (BMD) is a substantial risk of mortality in addition to fracture in hemodialysis patients. However, the factors affecting BMD are not fully determined. We conducted a single-center, cross-sectional study on 321 maintenance hemodialysis patients who underwent evaluation of femoral neck BMD using dual-energy X-ray absorptiometry from August 1, 2018, to July 31, 2019. We examined factors associated with osteoporosis defined by T-score of ≤  − 2.5, using logistic regression models. Median age of patients was 66 years, and 131 patients (41%) were diagnosed with osteoporosis. Older age, female, lower body mass index, diabetes mellitus, and higher Kt/V ratios were associated with higher osteoporosis risk. The only medication associated with lower osteoporosis risk was calcium-based phosphate binders (CBPBs) [odds ratio (OR), 0.41; 95% confidence interval (CI), 0.21–0.81]. In particular, CBPB reduced the osteoporosis risk within subgroups with dialysis vintage of ≥ 10 years, albumin level of < 3.5 mg/dL, active vitamin D analog use, and no proton pump inhibitor (PPI) use. In conclusion, CBPB use was associated with lower osteoporosis risk in hemodialysis patients. This effect might be partially attributable to calcium supplementation, given its higher impact in users of active vitamin D analogs or non-users of PPI, which modulate calcium absorption.

## Introduction

Osteoporosis is characterized by reduced bone mineral density (BMD) and microarchitectural deterioration of bone tissue, leading to bone fragility and a consequent increase in the risk of fractures^1,2^. Bone disease is a common complication in patients with chronic kidney disease (CKD), and such a condition is referred to as CKD mineral and bone disease (CKD–MBD). According to Kidney Disease Improving Global Outcomes (KDIGO) guidelines, CKD–MBD is a systemic disorder of mineral and bone metabolism due to CKD manifested by either one or a combination of the following: (1) abnormalities of calcium, phosphorus, intact parathyroid hormone (PTH), or vitamin D metabolism; (2) abnormalities in bone turnover, mineralization, volume, linear growth, or strength; or (3) vascular or other soft-tissue calcification. Data from the National Health and Nutrition Examination Survey have suggested that CKD and osteoporosis are highly coprevalent^3,4^ .

The incidence of fractures progressively increases according to CKD progression, and patients with CKD, particularly those with end-stage disease, have a high risk of fractures, which leads to unfavorable morbidity and mortality^5–12^. In the Dialysis Outcomes and Practice Patterns Study report including an international cohort of hemodialysis patients, the incidence of fractures was reported to be significantly higher for hemodialysis patients than for the general population, with a 3.7-fold increase in the unadjusted relative risk of mortality^8^. Furthermore, a more recent study including a Japanese cohort reported that the mortality rate after fractures was 4.8-fold higher in hemodialysis patients than in the general population^11^. Therefore, it is essential to prevent osteoporosis and fractures to improve the outcome of patients with ESKD.

Dual-energy X-ray absorptiometry (DXA) is a well-established tool for measuring BMD and strongly predicting the risk of fracture^13,14^. Accumulating evidence has revealed that DXA-based BMD predicts the risk of incident fractures in patients with advanced CKD, including hemodialysis patients, similar to that observed in the general population^15–18^. The latest KDIGO 2017 CKD–MBD guidelines recommend measurements of BMD in patients with advanced CKD. More recently, decreased DXA-based BMD was shown to predict a higher risk of overall mortality in patients with ESKD^12^. However, factors or medications that improve osteoporosis in this population have not been fully clarified because of the limited number of studies reported in the literature regarding factors associated with BMD in a large number of participants.

Therefore, the present study aimed to investigate clinical factors and medications, particularly including CKD–MBD-related medications, that are associated with BMD in maintenance hemodialysis patients using DXA.

## Material and methods

### Study design and participants

This is a single-center, cross-sectional study including 321 maintenance hemodialysis patients who underwent evaluations of femoral neck BMD using DXA from August 1, 2018, to July 31, 2019, at our dialysis center. Patients aged > 80 years or those who lacked routine laboratory data were excluded. All the patients received hemodialysis three times per week. The study protocol was approved by the ethics committee of Shuuwa General Hospital, and the study was performed in accordance with the Declaration of Helsinki guidelines regarding ethical principles for medical research involving human subjects. Informed consent was obtained from all participants after information about the study.

### Data collection

Baseline demographics and characteristics were recorded for each patient. Laboratory tests were performed in the first dialysis session of each week. We analyzed laboratory data measured within 1 month before DXA measurements, including serum albumin, sodium, calcium, phosphate, and urea nitrogen levels. In addition, we analyzed the plasma-intact PTH levels measured within 3 months before DXA measurements. Kt/V (Kt/V is a number used to quantify hemodialysis and peritoneal dialysis treatment adequacy) ratios were calculated using the single-pool Daugirdas formula. Geriatric nutritional risk index (GNRI) is a nutritional marker calculated by serum albumin level and body mass index (BMI)^19^. Percent creatinine generation rate (%CGR) is used as estimates of muscle mass and protein nutritional status^20^. Cardiovascular disease (CVD) was defined as any of the following: stroke, ischemic heart disease, congestive heart failure, or peripheral arterial disease. Hypertension was defined as a systolic blood pressure of ≥ 140 mmHg, a diastolic blood pressure of ≥ 90 mmHg, or taking antihypertensive agents. Diabetes mellitus was defined as hemoglobin A1C of ≥ 6.5%. Medication-related data and use of CBPBs, oral or IV-active vitamin D analogs, oral or IV calcimimetics, and PPIs were recorded. BMD was measured by DXA using a Horizon WI Bone Densitometer (Hologic Inc, Marlborough, Mass, USA). The results were expressed in terms of T-scores and standard deviation (SD) compared with healthy young sex-matched controls. Osteoporosis was defined as a T-score of ≤  − 2.5 according to the World Health Organization definition.

### Statistical analyses

Continuous data are shown as means with SD or medians with interquartile ranges (IQR), as appropriate. Categorical variables are expressed as numbers and percentages. Comparisons between the osteoporosis and non-osteoporosis groups were performed using the unpaired *t* test or Mann–Whitney *U* test for continuous variables and the chi-squared test for categorical variables, as appropriate. Multivariate logistic regression analyses were performed to evaluate factors associated with the risk of osteoporosis. Estimates of association were expressed as odds ratios (ORs) with the corresponding 95% confidence interval (CI). Subgroup analysis using multiple logistic regression analyses were performed to evaluate the effects of CBPBs on BMD under specific factors or backgrounds. Univariate and multivariate linear regression analyses were performed to examine the relevant factors associated with BMD. All statistical analyses were performed using EZR (Saitama Medical Center, Jichi Medical University, Saitama, Japan), which is a graphical user interface for R (The R Foundation for Statistical Computing, Vienna, Austria)^21^. *P* values of < 0.05 were considered statistically significant.

## Results

### Baseline characteristics

A total of 321 participants were enrolled in the study. Baseline characteristics and demographics of these patients are shown in Table 1. The median age was 66 years (IQR, 55–72 years), median dialysis vintage was 9 years (IQR, 5–17 years), 109 (34%) patients were women,138 (43%) were diagnosed with CVD, and 146 (46%) had diabetes mellitus. Osteoporosis was diagnosed in 131 (41%) patients. The median age was higher, the proportion of women was higher, and mean BMI was lower in the osteoporosis group than in the non-osteoporosis group. Median dialysis vintage and the prevalence of comorbidities such as CVD, hypertension, and diabetes mellitus were not significantly different in the two groups. With regard to laboratory data, mean serum phosphate levels and Kt/V ratios were higher in the osteoporosis group, whereas serum albumin, serum calcium, and serum intact PTH levels were not significantly different. The proportion of CBPB users was higher in the osteoporosis group, whereas the proportion of calcium-free phosphate binder, calcimimetics, and PPI users was not significantly different between the groups.

**Table 1:** Characteristics of the study participants.

Variable	Whole (n = 321)	Osteoporosis (n = 131)	Non-osteoporosis (n = 190)	*P* value
**Demographic**				
Age, years	66 (55–72)	68 (60–74)	63 (52–70)	< 0.001
Female, %	109 (34)	70 (53)	39 (21)	< 0.001
BMI, kg/m^2^	22.6 (19.5–25.0)	20.7 (18.1–23.1)	23.9 (20.8–26.7)	< 0.001
< 18.5, %	52 (16)	36 (28)	16 (8)	< 0.001
18.5–24.9, %	190 (59)	82 (63)	108 (57)	
≥ 25, %	79 (25)	13 (10)	66 (35)	
Dialysis vintage, year	9 (5–17)	10 (5–16)	8 (4–17)	0.3
Hypertension, %	319 (99)	130 (99)	189 (100)	1.0
Cardiovascular disease, %	138 (43)	64 (49)	74 (39)	0.1
Diabetes mellitus, %	146 (46)	62 (47)	84 (44)	0.7
**Laboratory data**				
Albumin, g/dL	3.6 ± 0.3	3.6 ± 0.4	3.6 ± 0.3	0.5
Sodium, mEq/L	137.9 ± 3.2	137.7 ± 32	138.0 ± 3.2	0.6
Calcium, mg/dL	8.8 ± 0.5	8.8 ± 0.5	8.8 ± 0.6	0.8
Phosphate, mg/dL	5.0 ± 1.1	4.7 ± 1.1	5.1 ± 1.1	0.001
PTH, pg/mL	191 (124–283)	195 (118–288)	190 (131–282)	1.0
Kt/V ratios	1.5 ± 0.2	1.6 ± 0.2	1.4 ± 0.2	< 0.001
**Medication**				
Ca-based P binders, %	251 (78)	91 (70)	160 (84)	0.003
Ca-free P binders, %	171 (53)	61 (47)	110 (58)	0.1
Active vitamin D analog, %	247 (77)	107 (82)	140 (74)	0.1
Calcimimetic, %	186 (58)	77 (59)	109 (57)	0.9
PPI, %	158 (49)	70 (53)	88 (46)	0.3

Data are shown as numbers (percentiles) for categorical variables and mean ± SD or median (interquartile range) for continuous variables. *P* < 0.05 was considered significant.

BMI, body mass index; Ca, calcium; P, phosphate; PPI, proton pump inhibitor; PTH, parathyroid hormone.

### Factors associated with osteoporosis in hemodialysis patients

To elucidate factors associated with low BMD, multivariate logistic regression analysis was performed. As shown in Table 2, older age, female sex, lower BMI, diabetes mellitus, lower serum phosphate level, and higher Kt/V ratios were associated with an increased risk of osteoporosis. Conversely, the use of CBPBs was associated with a decreased risk of osteoporosis after adjustment of potential confounders. The use of CBPBs, active vitamin D analogs, and PPI was not significantly associated with BMD.

**Table 2:** Factors associated with a risk of osteoporosis in maintenance hemodialysis patients.

Variable	Model 1		Model 2		Model 3	
	OR (95% CI)	*P* value	OR (95% CI)	*P* value	OR (95% CI)	*P* value
Age per 5 years	1.17 (1.04–1.32)	0.010	1.16 (1.02–1.31)	0.022	1.17 (1.02–1.34)	0.029
Female	4.26 (2.50–7.26)	< 0.001	4.88 (2.79–8.51)	< 0.001	3.24 (1.69–6.18)	< 0.001
**BMI, kg/m**^**2**^						
< 18.5	2.53 (1.26–5.10)	0.009	2.78 (1.35–5.73)	0.005	2.82 (1.25–6.38)	0.013
18.5–24.9	Reference		Reference		Reference	
≥ 25	0.26 (0.13–0.53)	< 0.001	0.23 (0.11–0.48)	< 0.001	0.30 (0.14–0.66)	0.003
Dialysis vintage per year			1.00 (0.97–1.03)	0.7	1.00 (0.97–1.04)	0.9
Hypertension			0.66 (0.02–20.90)	0.8	1.08 (0.03–35.80)	1.0
Cardiovascular disease			1.37 (0.79–2.38)	0.3	1.33 (0.72–2.46)	0.4
Diabetes mellitus			1.79 (0.96–3.32)	0.038	2.64 (1.31–5.34)	0.007
Albumin, g/dL					1.99 (0.80–4.97)	0.1
Sodium, mEq/L					1.02 (0.93–1.13)	0.6
Calcium, mg/dL					0.62 (0.36–1.06)	0.08
Phosphate, mg/dL					0.72 (0.54–0.95)	0.020
**PTH, pg/mL**						
Q1					1.38 (0.61–3.09)	0.4
Q2					Reference	
Q3					1.27 (0.56–2.85)	0.6
Q4					1.56 (0.69–3.55)	0.3
Kt/V ratios per 0.1					1.35 (1.13–1.61)	< 0.001
Ca-based P binders					0.41 (0.21–0.81)	0.011
Ca-free P binders					0.97 (0.54–1.74)	1.0
Active vitamin D analog					1.38 (0.65–2.94)	0.4
Calcimimetic					1.07 (0.59–1.92)	0.8
PPI					1.27 (0.71–2.27)	0.4

Multivariate logistic regression analysis for assessment of osteoporosis risk factors. Model 1, adjusted for age, sex, BMI. Model 2, age, sex, BMI, dialysis vintage year, hypertension, CVD, diabetes mellitus. Model 3, age, sex, BMI, dialysis vintage year, hypertension, CVD, diabetes mellitus, albumin, sodium, calcium, phosphate, iPTH, Kt/V ratios, CBPBs use, CBPB use, active vitamin D analogs use, calcimimetics use, and PPI use. Odds ratios (ORs) with the corresponding 95% confidence intervals (95% CIs). *P* < 0.05 was considered significant. BMI, body mass index; Ca, calcium; P, phosphate; PPI, proton pump inhibitor; PTH, parathyroid hormone.

We further examined the association between covariates and DXA-based BMD using multivariate linear regression analysis. Older age, female sex, lower BMI, diabetes mellitus, lower serum calcium level, and higher Kt/V ratios were associated with lower BMD (Table 3). The use of CBPBs was associated with a higher BMD, as revealed by univariate and multivariate analyses. In addition to these analyses using a T-score, we also examined the BMD values based on absolute measurement (g per cm^2^). As shown in the Supplementary Figure 1, the BMD values were greater in CBPB users than non-CBPB users particularly among male patients. The univariate and multivariate linear regression analyses revealed the factors associated with BMD (Supplementary Table 1), which was very similar to those observed in the analysis using a T-score (Table 3). CBPB use was significantly associated with greater BMD in a univariate linear regression model, and this association was also marginally significant after adjusting for multiple cofounders.

**Table 3:** Univariate and multivariate linear regression analyses examining the association between covariates and bone mineral density.

Variable	Univariate		Multivariate	
	Coefficient (95% CI)	*P* value	Coefficient (95% CI)	*P* value
Age per 5 years	− 0.139 (− 0.205 to − 0.073)	< 0.001	− 0.087 (− 0.147 to − 0.029)	0.004
Female	− 1.181 (− 1.466 to − 0.896)	< 0.001	− 0.781 (− 1.070 to − 0.491)	< 0.001
**BMI, kg/m**^**2**^				
< 18.5	− 1.153 (− 1.536 to − 0.771)	< 0.001	− 0.679 (− 1.027 to − 0.330)	< 0.001
18.5–24.9	Reference		Reference	
≥ 25	0.950 (0.622–1.278)	< 0.001	0.545 (0.234–0.855)	< 0.001
Dialysis vintage per year	− 0.001 (− 0.017–0.015)	0.9	0.007 (− 0.008–0.022)	0.4
Hypertension	0.077 (− 1.809–1.963)	0.9	− 0.032 (− 1.583–1.518)	1.0
Cardiovascular disease	− 0.318 (− 0.616 to − 0.020)	0.036	− 0.217 (− 0.478–0.044)	0.1
Diabetes mellitus	− 0.082 (− 0.380–0.220)	0.6	− 0.475 (− 0.767 to − 0.184)	0.002
Albumin, mg/dL	0.145 (− 0.306–0.596)	0.5	− 0.270 (− 0.658–0.118)	0.2
Sodium, g/dL	0.012 (− 0.035–0.059)	0.6	− 0.020 (− 0.060–0.020)	0.3
Kt/V ratios	− 0.289 (− 0.354 to − 0.223)	< 0.001	− 0.157 (− 0.230 to − 0.083)	< 0.001
Calcium, mg/dL	0.096 (− 0.180–0.372)	0.5	0.238 (0.004–0.471)	0.046
Phosphate, mg/dL	0.177 (0.045–0.309)	0.009	0.097 (− 0.018–0.211)	0.1
**PTH, pg/mL**				
Q1	Reference		Reference	
Q2	0.328 (− 0.013–0.669)	0.06	0.138 (− 0.211–0.487)	0.4
Q3	− 0.176 (− 0.516–0.163)	0.3	− 0.146 (− 0.500–0.208)	0.4
Q4	− 0.064 (− 0.409–0.280)	0.7	− 0.094 (− 0.463–0.275)	0.6
Ca-based P binders	0.412 (0.055–0.768)	0.024	0.303 (0.006–0.600)	0.046
Ca-free P binders	0.344 (0.049–0.639)	0.023	0.063 (− 0.191–0.317)	0.6
Active vitamin D analog	− 0.373 (− 0.723 to − 0.023)	0.037	− 0.199 (− 0.503–0.105)	0.3
Calcimimetic	− 0.080 (− 0.381–0.219)	0.6	− 0.138 (− 0.391–0.115)	0.2
PPI	− 0.104 (− 0.401–0.192)	0.5	− 0.018 (− 0.267–0.231)	0.9

Bone mineral density is based on a T-score that is the number of standard deviations compared with healthy young controls. β coefficients with the corresponding 95% confidence intervals (95% CIs). *P* < 0.05 was considered significant. BMI, body mass index; Ca, calcium; P, phosphate; PPI, proton pump inhibitor; PTH, parathyroid hormone; Q, quartile.

**Figure 1:**
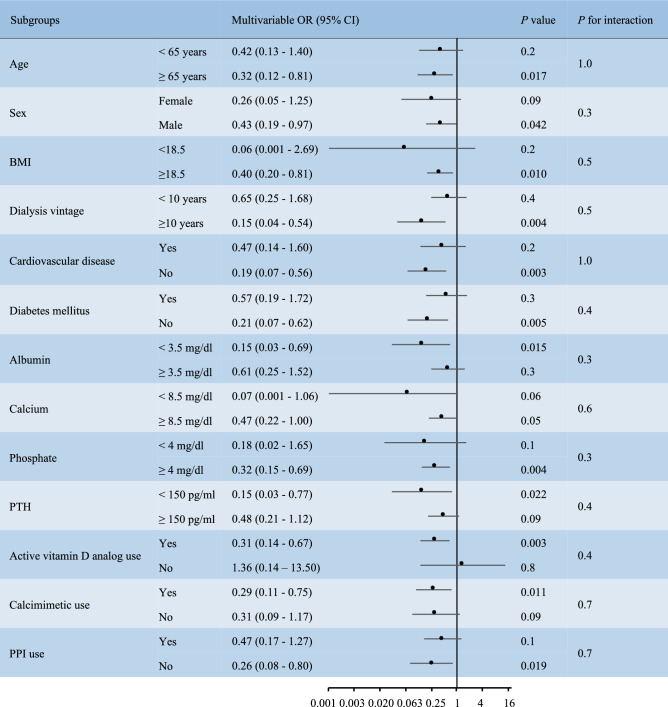
Evaluation of calcium-based phosphate binders’ effects on osteoporosis in various subgroups. Odds ratios (ORs) with the corresponding 95% confidence intervals (95% CIs). *P* < 0.05 was considered statistically significant. BMI, body mass index; PPI, proton pump inhibitor; PTH, parathyroid hormone.

### Subgroup analysis

To evaluate the benefits of CBPBs in treating osteoporosis in the specific population with ESKD, we used the association between CBPBs and the risk for osteoporosis in various subgroups based on multivariate logistic regression models. As shown in Fig. 1, CBPB was particularly associated with a decreased risk of osteoporosis in patients with longer hemodialysis vintage, lower serum albumin level, lower intact PTH level, and active vitamin D analog use as well as in non-CVD, non-diabetes mellitus, and non-PPI use patients.

To further determine if impact of CBPBs on osteoporosis risk is related to the patients’ nutritional status, we performed the additional subgroup analysis with the widely used nutritional status indicators GNRI and %CGR^19,20^. As shown in the Supplementary Figure 2, CBPBs were significantly associated with lower risk of osteoporosis in the lower GNRI or %CGR subgroups, which might reflect lower intake of dietary calcium.

## Discussion

This study investigated the association of multiple factors and medications with DXA-based BMD in maintenance hemodialysis patients and identified CBPB as a factor associated with a lower risk of osteoporosis in addition to the conventional risk factors for osteoporosis, including older age, female sex, lower BMI, diabetes mellitus, and higher Kt/V ratio. Moreover, we determined that the impact of CBPBs on BMD was greater in users of active vitamin D analogs or non-users of PPI, which modulate calcium absorption, suggesting that calcium supplementation by CBPBs increases BMD in patients with ESKD. To the best of our knowledge, this study is the first to report the beneficial effects of CBPBs on osteoporosis in patients with ESKD, providing new insights into osteoporosis treatment in this population.

The present study demonstrated that older age, female sex, lower BMI, and diabetes mellitus were correlated with lower BMD; these are well-established risk factors for osteoporosis in both the general population and patients with ESKD^22–29^. The association between higher Kt/V ratios and lower BMD demonstrated in the present study was unexpected, although the previous study including a large cohort of hemodialysis patients revealed that patients with fractures had higher Kt/V ratios^8^. In addition, we investigated the impact of medications, including CKD–MBD-related medications and PPI that is a recently identified risk factor for osteoporosis in adults and children^30^, on BMD and identified the previously unrecognized association between CBPB use and the decreased risk of osteoporosis.

It has long been unidentified whether CKD–MBD-related medications, including phosphate binders, modulate BMD and the subsequent risk of fracture in patients with ESKD. Recently, a prospective cohort study including 537 children with CKD reported that CBPBs reduced the risk of fractures^31^. A previous systematic review evaluating the effect of various phosphate binders on CKD–MBD-related outcomes of adult patients with CKD failed to conclude whether CBPB prevents fractures, possibly due to the enrollment of only one small randomized controlled trial based on 148 moderately ill patients with CKD^32,33^. A recent Korean population-based cohort study demonstrated that phosphate binders, including both CBPBs and non-CBPBs, reduced the risk of fractures in patients with ESKD^34^. The majority of phosphate binder users in this study were taking CBPBs, suggesting that the beneficial effect of phosphate binders was attributable to CBPBs and its favorable impact on BMD.

Calcium carbonate is the most used phosphate binder in patients with ESKD and is also used as a calcium supplement in the general population worldwide because of its cost efficiency and relatively high elemental calcium content. Although it is important to avoid excess calcium intake and prevent the increased risk of CVD events, kidney stones, and gastrointestinal symptom, adequate calcium intake is recommended for skeletal health in all age groups because long-term calcium deficiency can lead to osteoporosis and an increased risk of fracture^35–40^. However, several studies have indicated that the calcium intake in patients with CKD is basically low compared with the value recommended by dietary intake guidelines^41,42^. Thus, modest supplementation of calcium from foods, supplements, and calcium-containing medications, including CBPBs, is recommended for patients with CKD to achieve an estimated neutral calcium balance^43^. We speculated that calcium supplementation is among the major factors contributing to the increase in BMD in hemodialysis patients taking CBPBs, and the greater benefit of CBPBs among the subgroups with unfavorable nutritional status in our analyses support this speculation.

CBPBs were more likely to decrease the risk of osteoporosis, particularly in users of active vitamin D analogs and non-users of PPI, as revealed by our subgroup analysis. Vitamin D is also essential to bone health because it promotes intestinal calcium absorption. The efficacy of vitamin D alone or with concomitant use of calcium on osteoporosis and fractures has been examined in several trials in the general population. These trials revealed that vitamin D alone was insufficient but its concomitant use with calcium reduced the risk of fractures^44–48^. Considering these findings, we speculate that sufficient intake and adsorption of calcium is essential to prevent fractures, independent of the evidence of CKD. Furthermore, we demonstrated that CBPBs were less effective on BMD in PPI users.

Several studies, including a meta-analysis of case–control and cohort studies, have reported that PPIs are positively associated with an increased risk of fracture^30,49–53^. One possible explanation for this observation has been proposed: PPIs inhibit gastric acid, leading to impaired absorption of calcium^54–56^. Thus, this finding also supports the fact that calcium supplementation by CBPBs is partially responsible for increased BMD. We also demonstrated that CBPBs were effective in patients with reduced serum albumin level, lower BMI, and longer dialysis vintage. These results might be associated with the reduced nutrient intake, including calcium, in such patients. CBPBs were less effective in the CVD and diabetes mellitus groups. It is possible that a substantial proportion of these patients use loop diuretics, which increase calcium excretion.

Since excess of calcium intake might be harmful to CKD patients, the latest KDIGO guideline suggested restricting the dose of CBPBs among phosphate binders^57^. CBPB use is not associated with increase in mortality and vascular event risk compared with placebo in CKD patients. When compared with a calcium-free phosphate binder sevelamer, several clinical trials reported that CBPBs were associated with increased risk of vascular calcification^58–61^. However, the causal relationship between CBPBs and risk of mortality or cardiovascular events has yet to be determined, because “none of the studies provided sufficient dose threshold information about calcium exposure”^57^. Sevelamer is known to suppress vascular calcification due to mechanisms unrelated to calcium exposure such as improvement of the lipid profile, reduction of inflammation and oxidative stress^57,62,63^. The previous systematic review also demonstrated that risks of cardiovascular death, myocardial infarction, stroke, and coronary artery calcium score progression were not significantly different between a sevelamer and CBPBs, while risk of all-cause mortality was higher on CBPB use^32^. The findings in this study would provide novel insights into the association between CBPBs and CKD patients’ outcome, and indicate that CBPBs are potentially beneficial for bone health under avoidance of excessive calcium intake and adverse events. Further studies are needed to clarify the specific population who could receive therapeutic benefits of CBPBs.

The present study has several limitations. First, this was a single-center observational study. Second, because of the cross-sectional observational nature of this study, we could not establish causality. Third, data (such as serum bone alkaline phosphatase levels) regarding factors associated with bone metabolism were not available. Fourth, our dataset lacked diet inquiry or urinary calcium excretion that might be a nutritional marker of calcium intake, although urinary calcium may not reflect the dietary intake as the median dialysis vintage was nine years and 90% or more of the study participants were anuric or oliguric. Thus, we performed a subgroup analysis using the other nutritional status indicators GNRI and %CGR, and showed the higher impact of CBPBs on BMD under unfavorable nutritional status. Further investigations are warranted to establish the causal relationship between the use of CBPB and the prevention of osteoporosis and fractures.

## Conclusion

We have demonstrated that oral CBPB use was associated with a lower risk of osteoporosis in maintaining hemodialysis patients. The study findings might provide novel insights into osteoporosis treatment in patients with ESKD.

## Supplementary Information

Supplementary Information.
