# Supplementary material for: Calcium-based phosphate binder use is associated with lower risk of osteoporosis in hemodialysis patients
Source: Sci Rep. 2021 Jan 18;11:1648. doi: 10.1038/s41598-021-81287-4 (PMC7814124; doi:10.1038/s41598-021-81287-4)
Supplement: Supplementary file 1 — Supplementary Information. [file 41598_2021_81287_MOESM1_ESM.pdf]

Supplementary Information

Calcium-based phosphate binder use is associated with lower risk of osteoporosis in hemodialysis patients

Hiroko Hashimoto,[1] M.D., Ph.D.; Satomi Shikuma,[1] M.D.; Shintaro Mandai,[2]* M.D., Ph.D.; Susumu Adachi,[3] M.D., Ph.D.; Shinichi Uchida,[2] M.D., Ph.D.

[1]Department of Nephrology, Shuuwa General Hospital, 1200 Yaharashinden, Kasukabe, Saitama, 344-0035, Japan
[2]Department of Nephrology, Graduate School of Medical and Dental Sciences, Tokyo Medical and Dental University, 1-5-45 Yushima, Bunkyo, Tokyo, 113-8519, Japan
[3]Department of Cardiology, Shuuwa General Hospital, 1200 Yaharashinden, Kasukabe, Saitama, 344-0035, Japan

*Correspondence to: Shintaro Mandai

Department of Nephrology, Graduate School of Medical and Dental Sciences, Tokyo Medical and Dental University
1-5-45 Yushima, Bunkyo, Tokyo 113-8519, Japan
Tel: +81-3-5803-5214; Fax: +81-3-5803-5215; E-mail: smandai.kid@tmd.ac.jp

Supplementary Table 1. Univariate and multivariate linear regression analyses revealing the factors associated with bone mineral density in maintenance hemodialysis patients

	Univariate		Multivariate	
	Coefficient (95% CI)	*P* value	Coefficient (95% CI)	*P* value
Variable				
Age per 5 years	−0.014 (−0.022–−0.007)	<0.001	−0.009 (−0.014–−0.002)	0.007
Female	−0.135 (−0.165–−0.101)	<0.001	−0.093 (−0.123–−0.063)	<0.001
BMI, kg/m^2				
<18.5	−0.123 (−0.164– −0.083)	<0.001	−0.070 (−0.106–−0.033)	<0.001
18.5–24.9	Reference		Reference	
≥25	0.105 (0.070–0.014)	<0.001	0.063 (0.030–0.095)	<0.001
Dialysis vintage per year	0.0002 (−0.001–0.002)	0.8	0.001 (−0.000–0.002)	0.1
Hypertension	0.031 (−0.171–0.231)	0.8	0.024 (−0.139–0.186)	0.8
Cardiovascular disease	−0.032 (−0.064–−0.001)	0.045	−0.022 (−0.049–0.005)	0.1
Diabetes mellitus	−0.011 (−0.043–0.020)	0.5	−0.050 (−0.081–−0.020)	0.001
Albumin, g/dL	0.026 (−0.022–0.074)	0.3	−0.018 (−0.058–0.023)	0.4
Sodium, mEq/L	0.002 (−0.003–0.007)	0.5	−0.002 (−0.006–0.002)	0.4
Kt/V ratios	−0.313 (−0.383–−0.243)	<0.001	−0.165 (−0.242–−0.088)	<0.001
Calcium, mg/dL	0.0004 (−0.029–0.030)	1.0	0.017 (−0.008–0.041)	0.2
Phosphate, mg/dL	0.021 (0.007–0.035)	0.004	0.012 (−0.001–0.023)	0.07
PTH, pg/mL				
Q1	Reference		Reference	
Q2	0.031 (−0.005–0.067)	0.1	0.007 (−0.029–0.044)	0.7
Q3	−0.017 (−0.053–0.020)	0.4	−0.020 (−0.057–0.017)	0.3
Q4	−0.006 (−0.043–0.030)	0.7	−0.016 (−0.054–0.023)	0.4
Ca-based P binders	0.042 (0.004–0.080)	0.032	0.028 (−0.004–0.058)	0.08
Ca-free P binders	0.038 (0.007–0.070)	0.017	0.007 (−0.020–0.033)	0.6
Active vitamin D analog	−0.034 (−0.071–0.003)	0.07	−0.017 (−0.048–0.015)	0.3
Calcimimetic	−0.007 (−0.039–0.025)	0.6	−0.016 (−0.042–0.011)	0.3

Bone mineral density (gram per cm^2) is based on absolute measurement. β coefficients with the corresponding 95% confidence intervals (95% CIs). $P < 0.05$ was considered significant. BMI, body mass index; Ca, calcium; P, phosphate; PTH, parathyroid hormone; Q, quartile.

Supplementary Figure 1. Bone mineral density among male and female patients receiving maintenance hemodialysis with or without calcium-based phosphate binder use

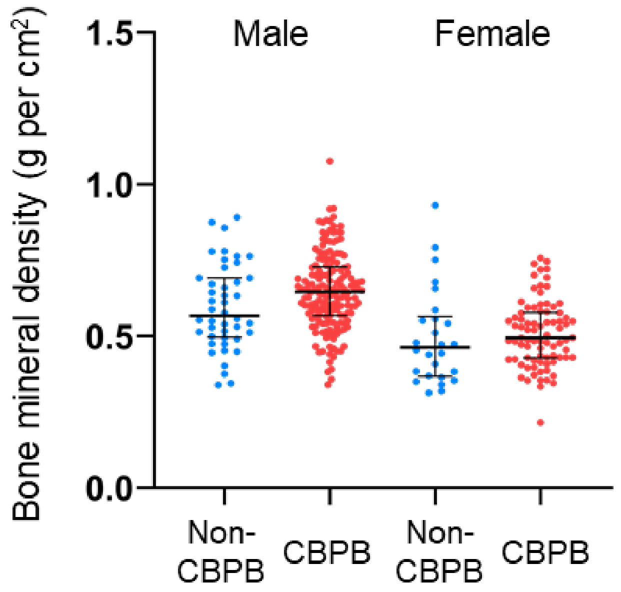

Bone mineral density (gram per cm^2) based on absolute measurement is described with a scatter plot showing the medians and interquartile ranges. CBPB, calcium-based phosphate binder.

Supplementary Figure 2. Impact of calcium-based phosphate binder use on osteoporosis risk among hemodialysis patients' subgroups associated with nutritional status

Subgroups		Multivariable OR (95% CI)	*P* value	*P* for interaction
GNRI	≤ 93.1	0.26 (0.09 - 0.72)	0.01	0.9
	> 93.1	0.39 (0.11 – 1.31)	0.1	
%CGR	≤ 102	0.15 (0.04 – 0.53)	0.003	0.5
	> 102	0.36 (0.12 – 1.08)	0.07	

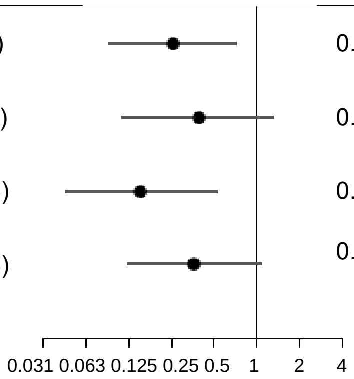

The median values of GNRI and %CGR were 93.1 and 102 in the study population, respectively. Odds ratios (ORs) with the corresponding 95% confidence intervals (95% CIs). $P < 0.05$ was considered statistically significant. GNRI, geriatric nutritional risk index; %CGR, Percent creatinine generation rate.
